# Expression of recombinant classical swine fever virus E2 glycoprotein by endogenous Txnip promoter in stable transgenic CHO cells

**DOI:** 10.1002/elsc.201900147

**Published:** 2020-07-01

**Authors:** Lei Feng, Li Chen, Junwen Yun, Xinglin Cao

**Affiliations:** ^1^ National Research Center of Engineering and Technology for Veterinary Biologicals Institute of Veterinary Immunology and Engineering, Jiangsu Academy of Agricultural Sciences Nanjing P. R. China; ^2^ Jiangsu Co‐innovation Center for Prevention and Control of Important Animal Infectious Diseases and Zoonoses Yangzhou P. R. China; ^3^ School of pharmacy Jiangsu University Zhenjiang P. R. China

**Keywords:** CHO‐dhfr–cells, dynamic expression, E2 protein, MTX, Txnip

## Abstract

As the main immunogen that could stimulate neutralized antibody in pigs, recombinant E2 protein of CSFV was expressed in CHO‐dhfr^−^cells driven by endogenous Txnip promoter from Chinese hamster. Different fragments of Txnip promoter were amplified by PCR from isolated genomic DNA of CHO cells and cloned into different expression vectors. Compared with CMV promoter, CHO‐pTxnip‐4‐rE2 (F12) cell clone with the highest yield of rE2 protein was established by random insertion of the expression cassette driven by 860 bp sequences of Txnip promoter. In combination with treatment of 800 nM MTX for copy amplification of inserted expression cassette, the dynamic expression profile of rE2 protein was observed. Then inducible expression strategy of balance between viable cell density and product yield was conducted by mixed addition of 0.1 mM NADH and 0.1 mM ATP in culture medium at day 3 of batch‐wise culture. It could be concluded that Txnip promoter would be a promising alternative promoter for recombinant antigen protein expression in transgenic cells.

**Abbreviations**
CSFV: classical swine fever virus
MTX: methotrexate.
rCHO cells: recombinant CHO cells
rE2 protein: recombinant E2 proteins
Txnip: thioredoxin‐interacting protein

## 1 INTRODUCTION

Classical swine fever (CSF) is a highly contagious multi‐systemic hemorrhagic viral and fatal disease of pigs. The etiological agent of CSF is classical swine fever virus (CSFV), which is a member of the genus *Pestivirus* of the *Flavivirade* family. The genome of CSFV consists of a single, positive‐stranded RNA of approximately 12.3 kb encoding for a polyprotein with 3898 amino acids, which could be cleaved into 12 mature viral proteins of four structural and eight nonstructural proteins [1]. The four structural proteins include nucleocapsid protein C and three envelope glycoproteins E^rns^, E1, and E2. E2 protein has been proven to be a most potent immunogen that could stimulate neutralized antibody in pigs [2, 3]. CSFV E2 protein has also been investigated in different expression systems, including baculovirus‐insect cells system [4], adenovirus [5], yeast [6, 7], plant [8], and even mammalian cells, like BHK21 cells [9] for subunit vaccine research and development.

Mammalian cell, especially Chinese hamster ovary (CHO) cell line, has been extensively served as host cell line for the production of therapeutic proteins with “native” mammalian glycosylation form. And the expression of antibody or cytokines is typically driven by a strong promoter, such as CMV promoter, SV40 promoter, EF‐1*α* promoter with constitutive expression pattern because of low cytotoxicity and efficient secretion [10]. But in some cases, negative effects of recombinant expression of exogenous protein caused by strong promoter in mammalian cells, such as viral antigen with lots of hydrophobic amino acids, on host mammalian cell growth and basic metabolism might be the main obstacle against achieving high productivity. Therefore, using inducible or dynamic promoter to express toxic protein could alleviate the negative effects. Temperature sensitive promoter S100a6 could achieve at least threefold increment of basal productivity after a temperature shift from 37 to 33°C [11]. Huong Le has also explored and identified several genes in CHO cells, such as *Mmp12*, *Txnip*, and *Serpinf1*, with an upswing expression pattern in the stationary phase relative to the exponential phase, based on time‐series transcriptome data [12]. Furthermore, dynamic expression of fructose transporter (GLUT5) in CHO cells under the control of Txnip promoter resulted in more moderated sugar consumption, better growth characteristics, and higher product yielding, which meant that time dynamic expression trend could be served for not only transgenes in cell engineering but exogenous and cytotoxic protein expression to allow trade‐offs between cell growth and product yield and to avoid undesired “chaos” in cell metabolism [13].

Therefore, in this work, we developed new stable transgenic recombinant CHO (rCHO) cells with the dynamic Txnip promoter to express recombinant CSFV E2 protein. Some inducible expression strategies had also been explored in suspension cultured mode.

## 2 MATERIALS AND METHODS

### 2.1 Cells

CHO‐dhfr^–^cells (ATCC^®^ CRL‐9096™) were cultured in Iscove's modified Dulbecco's medium (IMDM, Gibco) supplemented with 4 mM l‐glutamine, 1.5 g/L sodium bicarbonate, 0.1 mM hypoxanthine, 0.016 mM thymidine (H/T, Gibco, 11067030), and 10% FBS (Gibco, 10099–133) at 37°C in a 5% CO_2_ atmosphere.

**Practical application**

The current work demonstrated that endogenous promoter from Chinese hamster, Txnip promoter, could successfully be applied to express recombinant CSFV E2 protein, which could cause ER stress and NLPR3 inflammasome in host cells infected by CSFV. The dynamic expression pattern and inducible expression strategy achieved in this work showed that normal biomass increasing and basic metabolism of recombinant CHO cells should be considered as a prerequisite for recombinant protein expression, especially viral antigen with lots of hydrophobic amino acids and cytotoxicity. This expression strategy and CHO‐pTxnip‐4‐rE2 cell clone have been applied for patent (201810620594.4) for CSFV subunit vaccine development. Furthermore, specific integration of rE2 coding sequences at the downstream of endogenous Txnip promoter has been performed in different mammalian cells, such as CHO cells, PK15 cells, and ST cells, by CRISPR‐Cas9 system in our laboratory, showing the same dynamic expression pattern (data not shown in this work).

### 2.2 Construction of plasmids for expression of recombinant CSFV‐E2 protein

The codon‐optimized DNA sequences encoding E2 protein with several amino acid mutations according to the CSFV epidemiologic research in China, such as D705N, L709P, G713E, N723S, and S779A, were chemically synthesized and cloned into the pUC57 plasmid. The pUC57‐rE2 plasmid was digested with *EcoRI* and *HindIII*. The target DNA fragment was cloned into the *EcoRI* and *HindIII* sites of the expression vector pcDNA3.1(+) to generate pcDNA3.1‐rE2. Then, the codon‐optimized DNA sequences of DHFR expression cassette including murine β‐globin transcriptional regulation unit, DHFR coding sequences, bGH polyA signal sequences were cloned into pcDNA3.1‐rE2 vector by two restriction enzyme sites *BstZ17I* and *PciI* to generate pcDNA3.1‐rE2‐dhfr vector, designated as pCMV‐rE2. This vector contains the neomycin resistance gene, which confers resistance to G418. DNA fragments of Txnip promoter were amplified from the isolated genomic DNA of CHO‐dhfr^–^cells by a set of primers as follows,

P1: GGACGCGTGCTCCTAGCCCGGCAGCTATATAA,
P2: GGACGCGTGGATTGGTCGGAGGCCTGGTA,
P3: GGACGCGTTGGATGGGGTTCAGGGTCGCC,
P4: GGACGCGTTAGACATGCAACGGGAAGACACCG,
P5: GGGCTAGCGATTGGGTTCAGCGGGTTCCAG.

**FIGURE 1:**
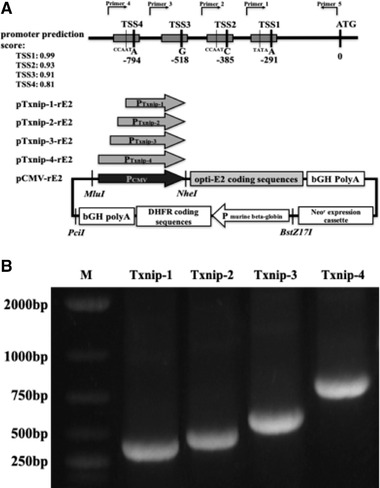
Txnip promoter TSS prediction, PCR amplification and expression vectors construction. (A) Schematic illustration of different Txnip promoter fragments and expression vectors construction. According to the promoter prediction score, four different promoter fragments were amplified by PCR and used to construct different expression vectors. (B) Four PCR products of different Txnip promoter sequences. M: DNA marker, Txnip‐1–4: PCR products amplified with different pairs of primers as shown in materials and methods section

PCR products of 339, 434, 592, and 860 bp were illustrated as shown in Figure 1. Followed with checking of sequencing data, different DNA fragments of Txnip promoter were cloned into pCMV‐rE2 vector by swapping the DNA fragment of CMV promoter to generate different pTxnip‐rE2 vectors with *MluI* and *NheI*, named as pTxnip‐1‐rE2, pTxnip‐2‐rE2, pTxnip‐3‐rE2, and pTxnip‐4‐rE2. These vectors, including pCMV‐rE2 and different pTxnip‐rE2 vectors, were used to transfect CHO‐*dhfr* ^–^cells to generate recombinant CHO cell clones with expression of recombinant E2 protein of CSFV.

### 2.3 Establishment of stable CHO cell lines producing recombinant E2 protein

On the day before transfection, CHO‐*dhfr* ^–^cells were prepared and inoculated in the six‐well plate at the initial cell density of 1 × 10^6^ cells/mL. CHO cells with monolayer were transfected with the pCMV‐rE2 and different pTxnip‐rE2 vectors respectively using branched 25 kDa polyethylenimine (PEI, Sigma–Aldrich, 764604). The transfection procedure based on PEI was performed as previously described [14, 15]. One day after the transfection, the different transfected cell groups were digested with trypsin and split into 10‐cm plates respectively in growth media with dialyzed FBS and without H/T addition for transgenic cells forming, followed with two rounds drug selection of 800 and 1200 μg/mL G418 (Roche, 04727878001). Then, G418‐resistant cells from different transfected groups were sorted into 96‐well plates to generate single cell clone in each well by flow cytometry respectively. The total intracellular protein were extracted by membrane and cytosol protein extraction kit (Beyotime, P0033) and recombinant E2 protein productivity in cytoplasm and cytomembrane were compared in all single cell clones by measuring the optical density at 450 nm using a CSFV Ag ELISA kit (Median Diagnostic Inc., Korea) according to the manual instruction.

The selected top five single cell clones with the highest expression level of rE2 protein from each transfected cell pool were transferred into growth media (without H/T) supplemented with 200–1200 nM methotrexate (MTX, Sigma–Aldrich, M8407) step by step to increase the copies of integrated vector including recombinant CSFV E2 protein and DHFR expression cassettes. The productivity of recombinant E2 protein of each cell clone after MTX treatment was measured as OD 450 nm and compared to screen out the best cell clone. To measure cell growth ability and recombinant E2 expression level of each cell clone, 10 cell clones from CHO pCMV‐rE2 cell pool (A4, A11, D3, F6, and G7) and CHO pTxnip‐4‐rE2 cell pool (B6, D2, D9, F12, and G5) were adapted into CHO serum free media (SFM, ToCell biotech, 701050) for suspension culture in shaking flasks (50 mL) on Kühner shaker at 180 rpm. Viable cell density of each cell clone was monitored by a Fisher Scientific hemocytometer with the trypan blue dye‐exclusion method and rE2 expression levels were assayed by ELISA method as mentioned above during a whole batch‐wise culture of 6 days.

### 2.4 Development of rCHO cells in serum free media for rE2 production and physiological characterization

The cell clone with the highest productivity of recombinant CSFV E2, CHO‐pTxnip‐4‐rE2 cell clone (F12), was designated as the target cell clone for recombinant E2 protein production in suspension cultured mode. CHO‐pCMV‐rE2 (A11) and parental CHO cells were conducted the same process as control. In 6 days batch‐wise suspension culture performed in 1 L shaking flask with 550 mL SFM, recombinant CSFV E2 protein generated in different CHO cells was prepared by collecting cell pellets spun from 1 mL cell culture and assayed by ELISA as mentioned above. Transcriptional trends of rE2 protein and endogenous Txnip in different CHO cells were also illustrated as delta Ct value (Ct*_ß_*_‐actin_ ‐ Ct_rE2_ or Ct*_ß_*_‐actin_ ‐ Ct_Txnip_) tested by quantitative real‐time PCR assay during the whole batch cultural process. The RNA samples were prepared using RNeasy Mini Kit (Qiagen, Cat.74104) and treated with DNase I before reverse transcription to remove genome DNA. The cDNA was synthesized by using QuantiNova Reverse Transcription Kit (Qiagen, Cat.205413) and qRT‐PCR was performed using QuantiFast SYBR Green PCR Kit (Qiagen, Cat.204054) in the LightCycler 480 II (Roche, Germany). The sequences of primers used in this process were listed as follows, rE2: F‐CTGATCGGCAACACCAC, R‐TACTCGCCCTTCAGCAT, Txnip: F‐CAGTGCAAACAGACCTTGGA, R‐AAGGAAAGCCTTCACCCAGT, *ß*‐actin: F‐GTCGTACCACTGGCATTGTG, R‐AGGGCAACATAGCACAGCTT.

Viable cell density of different CHO‐rE2 cells and parental CHO cells was monitored during the whole suspension cultural process by a Fisher Scientific hemocytometer with the trypan blue dye‐exclusion method. The concentrations of glucose, glutamine, lactate in the medium were assayed using Nova 400 instrument.

### 2.5 Induction of recombinant E2 protein and endogenous Txnip expression by addition of small molecules

To test the impacts of NADH and ATP on induction of rE2 and endogenous Txnip expression, 0.1 mM NADH and 0.1 mM ATP were respectively supplemented in SFM to regulate target proteins expression in CHO‐pTxnip‐4‐rE2 (F12) cells. Different cell pellet samples were collected at different time points after addition of NADH and ATP. The reverse transcription process and quantitative real‐time PCR assay were performed as mentioned above. Beta‐actin gene of CHO cells as loading control was used for the relative quantification. The fold changing values of target genes compared with the samples of CHO‐pTxnip‐4‐rE2 cells without NADH or ATP induction were calculated by 2^−ΔΔCt^ method [16, 17]. Each reaction was performed in triplicates and results were plotted as average ± SD of the mean. And with the mixed addition of 0.1 mM NADH and 0.1 mM ATP at days 2, 3, and 4 of batch‐wise suspension culture performed in 1 L shaking flask with 550 mL SFM respectively, rE2 yield and viable cell density of F12 cells were assayed and compared with F12 cells without induction.

### 2.6 Statistical analysis

In each data set, *P*‐values were determined by *t*‐test using GraphPad Prism 6.0. *P*‐values < 0.05 were considered significantly.

## 3 RESULTS

### 3.1 Construction of expression vectors

According to the promoter prediction score, different promoter fragments of *Txnip* in CHO cells, designated as Txnip 1–4, were amplified by PCR with different pairs of primers. The predicted information of Txnip promoter and PCR products of different fragments were illustrated in Figure 1A,B. After different PCR fragments were swapped for CMV promoter in the expression vector pCMV‐rE2 respectively by sub‐cloning with *MluI* and *NheI*, different expression vectors were completed for this work.

### 3.2 Establishment of stable cell clones with rE2 expression

**FIGURE 2:**
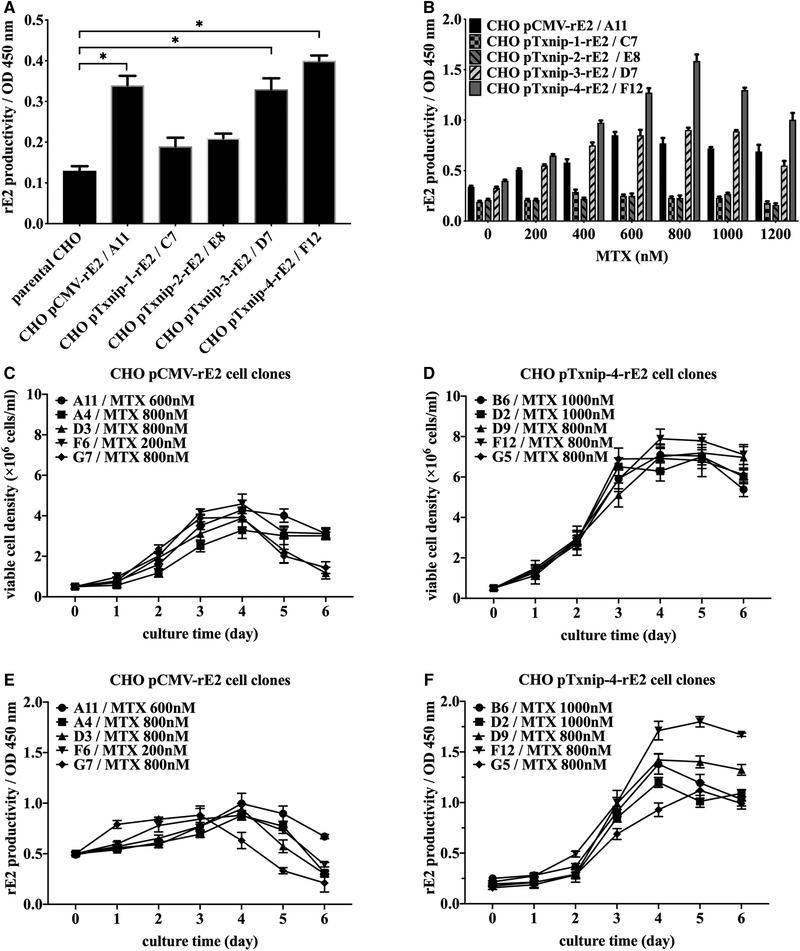
Development and selection of target cell clone. (A) Initial selection of recombinant CHO cell clones with transgenic expression of recombinant CSFV‐E2 protein driven by different promoters after second G418 screening. ^*^ *P* < 0.05 was considered statistically significant compared with parental CHO cells. (B) Effects of methotrexate on recombinant E2 productivity in five specific cell clones from each transfected cell pool with the increasing of MTX concentration from 200 to 1200 nM (B). (C and D) Measurement of viable cell density of top five cell clones from CHO pCMV‐rE2 cell pool and CHO pTxnip‐4‐rE2 cell pool respectively each day. (E and F) Measurement of rE2 expression level of top five cell clones from CHO pCMV‐rE2 cell pool and CHO pTxnip‐4‐rE2 cell pool respectively each day. Results were presented as average values ± SD from three independent samples of each cell clone

Top five cell clones from each transfected cell pool with the highest expression level of rE2 are listed in Table 1. Before MTX treatment, the cell clone with the highest expression level of each cell pool, such as CHO‐pCMV‐rE2‐A11, CHO‐pTxnip‐1‐rE2‐C7, CHO‐pTxnip‐2‐rE2‐E8, CHO‐pTxnip‐3‐rE2‐D7, and CHO‐pTxnip‐4‐rE2‐F12, were compared for the initial level screening, as shown in Figure 2A. Fragment Txnip‐1 and Txnip‐2 as promoter caused much lower expression level of rE2 protein than other experimental groups, which indicated that two fragments of Txnip‐1 and Txnip‐2 might not contain full sequences of Txnip promoter. However, cell clones with Txnip‐3, Txnip‐4, and CMV promoter could significantly express rE2 as the initial level before MTX treatment.

**TABLE 1:** MTX treatment on top five cell clones with highest rE2 expression level from each vector transfected cell pool

		Recombinant E2 productivity/OD 450 nm^a^						
Cell pool	Top 5 clones after second G418 screening	MTX 0 nM	MTX 200 nM	MTX 400 nM	MTX 600 nM	MTX 800 nM	MTX 1000 nM	MTX 1200 nM
CHO pCMV‐rE2	A4	0.28 ± 0.01	0.44 ± 0.02	0.59 ± 0.04	0.67 ± 0.06	0.76 ± 0.06	0.59 ± 0.06	0.47 ± 0.05
	A11	0.34 ± 0.02	0.51 ± 0.01	0.58 ± 0.03	0.85 ± 0.05	0.77 ± 0.05	0.72 ± 0.01	0.69 ± 0.07
	D3	0.27 ± 0.01	0.37 ± 0.05	0.51 ± 0.01	0.66 ± 0.05	0.69 ± 0.05	0.49 ± 0.04	0.42 ± 0.03
	F6	0.31 ± 0.01	0.68 ± 0.03	0.61 ± 0.02	0.53 ± 0.04	0.41 ± 0.05	0.41 ± 0.06	0.31 ± 0.04
	G7	0.30 ± 0.02	0.49 ± 0.01	0.59 ± 0.01	0.63 ± 0.07	0.67 ± 0.04	0.67 ± 0.07	0.39 ± 0.03
CHO pTxnip‐1‐rE2	B4	0.16 ± 0.01	0.19 ± 0.01	0.19 ± 0.01	0.20 ± 0.02	0.19 ± 0.02	0.20 ± 0.01	0.21 ± 0.01
	C7	0.19 ± 0.02	0.21 ± 0.01	0.29 ± 0.02	0.25 ± 0.01	0.23 ± 0.01	0.23 ± 0.01	0.18 ± 0.02
	D9	0.15 ± 0.01	0.16 ± 0.01	0.17 ± 0.02	0.18 ± 0.01	0.16 ± 0.01	0.16 ± 0.02	0.17 ± 0.02
	E3	0.16 ± 0.01	0.16 ± 0.02	0.18 ± 0.01	0.20 ± 0.01	0.21 ± 0.02	0.19 ± 0.01	0.18 ± 0.01
	E6	0.14 ± 0.01	0.15 ± 0.02	0.16 ± 0.02	0.16 ± 0.01	0.17 ± 0.03	0.16 ± 0.02	0.16 ± 0.01
CHO pTxnip‐2‐rE2	B2	0.19 ± 0.01	0.20 ± 0.01	0.21 ± 0.02	0.22 ± 0.01	0.19 ± 0.01	0.20 ± 0.01	0.17 ± 0.01
	B6	0.19 ± 0.01	0.21 ± 0.02	0.23 ± 0.03	0.26 ± 0.03	0.23 ± 0.02	0.20 ± 0.02	0.18 ± 0.01
	C11	0.18 ± 0.01	0.19 ± 0.02	0.17 ± 0.01	0.16 ± 0.02	0.16 ± 0.02	0.16 ± 0.01	0.16 ± 0.02
	D8	0.18 ± 0.01	0.18 ± 0.03	0.18 ± 0.03	0.20 ± 0.03	0.21 ± 0.02	0.20 ± 0.01	0.18 ± 0.01
	E8	0.21 ± 0.01	0.21 ± 0.01	0.22 ± 0.01	0.25 ± 0.02	0.23 ± 0.02	0.27 ± 0.03	0.16 ± 0.02
CHO pTxnip‐3‐rE2	B6	0.30 ± 0.01	0.33 ± 0.02	0.47 ± 0.04	0.66 ± 0.05	0.77 ± 0.08	0.67 ± 0.07	0.57 ± 0.06
	C7	0.31 ± 0.01	0.48 ± 0.05	0.58 ± 0.07	0.68 ± 0.07	0.74 ± 0.09	0.88 ± 0.09	0.58 ± 0.03
	D7	0.33 ± 0.03	0.55 ± 0.01	0.75 ± 0.03	0.85 ± 0.05	0.91 ± 0.02	0.89 ± 0.06	0.55 ± 0.05
	F9	0.29 ± 0.01	0.37 ± 0.05	0.67 ± 0.04	0.36 ± 0.05	0.37 ± 0.02	0.27 ± 0.03	0.17 ± 0.02
	G9	0.28 ± 0.01	0.39 ± 0.04	0.48 ± 0.05	0.78 ± 0.08	0.87 ± 0.10	0.58 ± 0.07	0.48 ± 0.04
CHO pTxnip‐4‐rE2	B6	0.37 ± 0.01	0.59 ± 0.04	0.67 ± 0.09	0.86 ± 0.09	0.97 ± 0.11	1.27 ± 0.13	1.13 ± 0.12
	D2	0.39 ± 0.01	0.47 ± 0.05	0.58 ± 0.07	0.78 ± 0.12	0.89 ± 0.08	1.08 ± 0.11	1.01 ± 0.08
	D9	0.35 ± 0.01	0.58 ± 0.03	0.67 ± 0.08	0.96 ± 0.11	1.36 ± 0.12	1.16 ± 0.09	0.96 ± 0.09
	F12	0.41 ± 0.02	0.65 ± 0.04	0.98 ± 0.03	1.27 ± 0.04	1.59 ± 0.09	1.29 ± 0.07	1.01 ± 0.06
	G5	0.34 ± 0.01	0.51 ± 0.05	0.67 ± 0.04	0.76 ± 0.04	0.97 ± 0.08	0.77 ± 0.07	0.57 ± 0.06

a Results are presented as average values ± SD from three samples of each cell clone.

After approximately 8‐week MTX treatment, the expected correlation between uprising of rE2 expression and increased resistance to MTX from 200 to 800 nM was observed in cell clones from CHO pCMV‐rE2, CHO pTxnip‐3‐rE2, and CHO pTxnip‐4‐rE2 cell groups, as listed in Table 1 and shown in Figure 2B. However, when the recombinant cell clones were exposed to the much higher MTX concentration at 1000 or 1200 nM, the expression level of rE2 protein decreased in some cell clones instead, implying an inherent restriction in the extent of gene amplification in the DHFR‐MTX system.

Viable cell density and rE2 protein expression level of 10 cell clones from CHO pCMV‐rE2 cell pool and CHO pTxnip‐4‐rE2 cell pool were compared in 50 mL shaking flasks respectively, as shown in Figure 2C–F. Cell clones from pTxnip‐4‐rE2 transfected cell pool could generally achieve higher viable cell density and rE2 expression level after 3 days suspension culture than cell clones from CHO pCMV‐rE2 cell pool. CHO‐pTxnip‐4‐rE2 cell clone (F12) at 800 nM MTX reached the highest expression level of rE2 and was picked up and designated as the target cell clone for next step. CHO‐pCMV‐rE2 cell clone (A11) acquired the maximum rE2 yield at 600 nM MTX and then was conducted on the same process as control.

### 3.3 Expression pattern of rE2 protein in stable cell clones driven by two promoters

**FIGURE 3:**
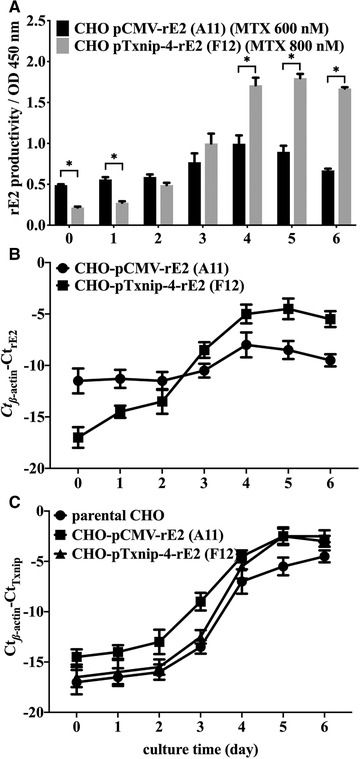
Expression patterns of recombinant CSFV‐E2 protein caused by different promoter within 6 days batch‐wise culture process. (A) Expression profile of rE2 protein in CHO pTxnip‐4‐rE2 (F12) cells CHO pCMV‐rE2 (A11) cells cultured in 1 L shaking flasks. Statistically significant differences between two cells were analyzed by *t*‐test (^*^ *P* < 0.05). (B) The gradual upward trend of mRNA level of rE2 driven by Txnip‐4 promoters in CHO pTxnip‐4‐rE2 (F12) cells. (C) Dynamic trend of endogenous Txnip transcription in parental CHO cells, CHO pTxnip‐4‐rE2 (F12) cells CHO pCMV‐rE2 (A11) cells. Results were presented as average values ± SD from three independent cultures of each cell clone

As shown in Figure 3A, rE2 expression level in F12 cells maintained low level on the first 2 days of batch culture and significantly increased much higher level at the later phase of batch culture with 800 nM MTX, then decreased slightly at the end of culture. Nevertheless, A11 cells obtained significantly higher rE2 productivity than that of F12 cell clone on the initial 2 days, while the maximum rE2 expression level at day 4 of batch culture was significantly lower than that of F12 cell clone. The mRNA changing trend of rE2 in two recombinant CHO cells were also monitored during the whole cultural process as showed in Figure 3B. Txnip‐4 promoter could achieve typical dynamic expression pattern in F12 cells. The transcriptional levels of endogenous Txnip in different CHO cells were also tested (Figure 3C), showing the expected upswing dynamics from the mid‐phase to the late‐phase. And interestingly in A11 cells, the expression of endogenous Txnip was higher on the first 3 days than two other cells, which might be attributed to the viral antigen synthesis burden driven by a strong promoter.

### 3.4 Physiological characteristics of recombinant CHO cells for rE2 expression

**FIGURE 4:**
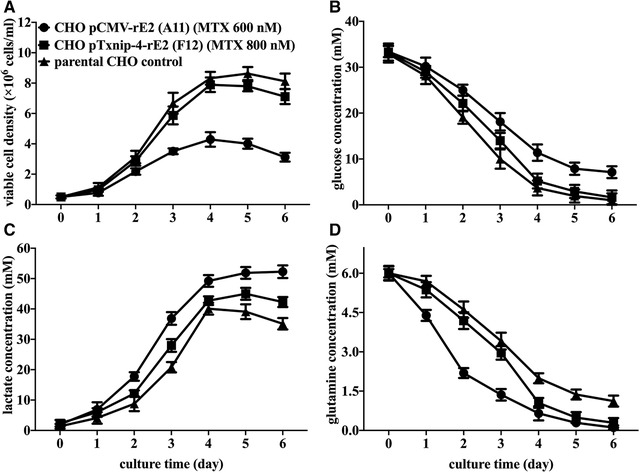
Comparison of time‐course profile of viable cell density (A), glucose (B), lactate (C), and glutamine (D) concentration in parental CHO cells, CHO‐pCMV‐rE2 (A11) cells, and CHO‐pTxnip‐4‐rE2 (F12) cells within 6 days batch‐wise culture process. Results were presented as average values ± SD from three independent cultures of each cell clone

Two recombinant cells (F12 and A11) and parental CHO cells were inoculated at the same initial cell density in suspension cultured mode. The resulting cell growth curve, glucose, lactate, glutamine concentration profiles of different CHO cells are shown in Figure 4. Although exponential cell growth was observed in three CHO cells, F12 cells reached its maximum viable cell density at the fourth day with viable cell density of 7.89 × 10^6^ cells/mL without significant difference with parental CHO cells (8.32 × 10^6^ cells/mL). Nevertheless, the viable cell density of A11 cells was significantly lower than two other cells. The lower biomass might be responsible for the lower rE2 expression level. As for glucose consumption and lactate production (Figure 4B and C), F12 cells and parental CHO cells ceased growth once glucose was almost depleted after 4 days culture and the switch of lactate generation to consumption was only observed in these two cells at the end of the culture. While the glucose consumption of A11 cells was less than that of other two cells during the whole batch culture process. By contrast, the glutamine utilization in A11 cells was much faster and higher than that in other two cells (Figure 4D). Considering the continuous accumulation of lactate during the whole culture, it could be speculated that exogenous viral antigen expression driven by a strong promoter might require much more metabolic flux of the glutaminolytic pathway to supply more NADPH and fatty acids.

### 3.5 Inducible expression of rE2 protein in rCHO cells

**FIGURE 5:**
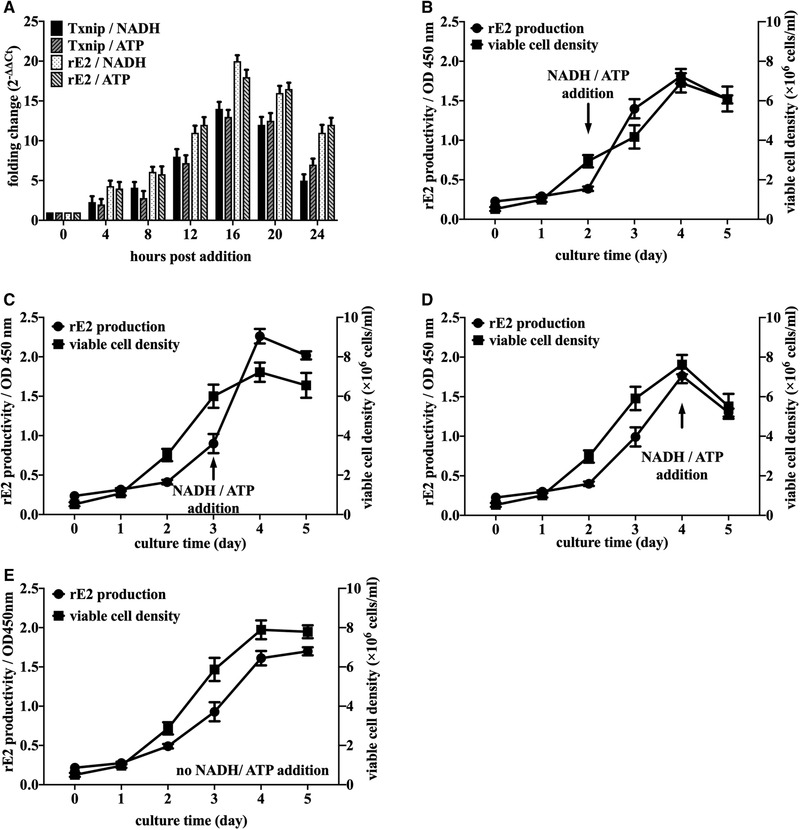
Inducible expression strategy of recombinant CSFV‐E2 protein by addition of adenosine‐containing molecules in single cell suspension culture of CHO pTxnip‐4‐rE2 (F12) cells. (A) Time course profile of mRNA changing folds induced by 0.1 mM NADH and 0.1 mM ATP on endogenous Txnip and recombinant CSFV‐E2 in CHO pTxnip‐4‐rE2 (F12) cells. (B–D) Comparison of viable cell density and rE2 productivity by mixed addition of 0.1 mM NADH and 0.1 mM ATP at days 2–4 of batch culture in CHO‐pTxnip‐4‐rE2 (F12) cells, respectively. (E) Viable cell density and rE2 productivity of CHO‐pTxnip‐4‐rE2 (F12) cells without induction. Results are presented as average values ± SD from three independent experiments

Since adenosine‐containing molecules could ubiquitously induce Txnip expression in different cells or tissues [18], induction of rE2 expression driven by Txnip promoter was performed by addition of NADH and ATP in cultural medium. As shown in Figure 5A, the time course of inducible expression of rE2 in F12 cells was illustrated at mRNA level with about 4–20‐fold increasing from 4 to 16 h after addition of 0.1 mM of NADH or ATP, compared with non‐induced F12 cells. As expected, the increased endogenous Txnip expression at the transcriptional level was also observed. After 16 h post addition, the induction effect on target genes decreased apparently. According to the time course of induction effects of NADH and ATP, mixed addition of 0.1 mM NADH and 0.1 mM ATP at days 2–4 of batch culture was respectively conducted to assay rE2 productivity and viable cell density in suspension cultured mode. Compared with F12 cells without adenosine‐containing molecules induction (Figure 5E), 0.1 mM NADH and 0.1 mM ATP mixed addition at the second or third day of batch culture could increase the production of rE2 protein during the next 24 ho, but showing slight inhibition in cell growth (Figure 5B,C). However, at the fourth day of batch culture, mixed addition of NADH and ATP could not increase rE2 productivity efficiently and it might be attributed to the lower residue glucose extracellularly, which implied that the induction of Txnip transcription by adenosine‐containing molecules might be tightly correlated with extracellular glucose level or be a glucose‐dependent manner.

## 4 DISCUSSION

The thioredoxin (Trx) system, which is composed of NADPH, thioredoxin reductase (TrxR), and thioredoxin, is a key antioxidant system in defense against oxidative stress through its disulfide reductase activity regulating protein dithiol/disulfide balance [19]. Thioredoxin‐interacting protein (Txnip, also termed VDUP1 for vitamin D upregulated protein or TBP2 for thioredoxin‐binding protein), was originally discovered by its strong regulation by vitamin D. Txnip has been found to regulate the cellular reduction–oxidation (redox) state by binding to and inhibiting thioredoxin in a redox‐dependent manner [20]. Recently, it has also been testified that Txnip could interact with NLRP3 and lead to NLRP3 inflammasome activation in a ROS‐sensitive manner, indicating that Txnip might play a major role in the convergence of the multiple signaling pathways that contribute to oxidative stress‐related disorders [21, 22, 23, 24].

**FIGURE 6:**
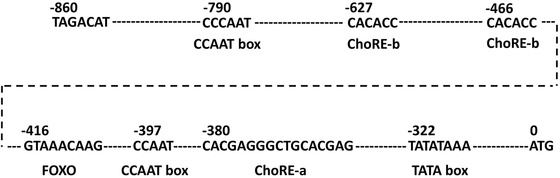
Sequencing results of Txnip‐4 fragment from Chinese hamster and potential cis‐regulatory elements in this region. Numbers indicate the upstream distance to the starting codon, ATG

Txnip, as a potent growth suppressor, induced G1 arrest in the cell cycle by its overexpression in association with the induction of p16 gene expression in HTLV‐I infected MT‐2 cells [25]. Expression of Txnip has also been identified as downregulation in several tumors, lymphoma, and melanoma metastasis [26]. The Txnip expression was then testified to be induced by glucose extracellularly, which could be mediated by carbohydrate‐response elements (ChoREs) and associated transcription factors Max‐like protein X (MLX) and ChoRE‐binding protein (termed as Mondo) [27]. And generally, two CCAAT boxes were found in Txnip promoter sequences from different species, which could recruit nuclear factor Y (NF‐Y) to the Txnip promoter. Not only that, the occupancy of the Txnip promoter by NF‐Y might be a prerequisite for efficacious recruitment of Mondo/MLX to ChoREs [28]. Checking the sequences of Txnip‐4 promoter from Chinese hamster, several regulatory elements were illustrated in this region, as shown in Figure 6, such as tandem ChoREs (ChoRE‐a and ChoRE‐b), FOXO, two CCAAT motifs, and TATA box. According to the results of this work, fragments Txnip‐1 (339 bp, only containing TATA box) and Txnip‐2 (434 bp, without ChoRE‐b elements) could not drive transcription and expression of the target protein because of lack of basic functional elements. And Txnip‐3 (592 bp) with one ChoRE‐b sequence and downstream elements could generate approximately 70–80% promoter function. Containing the whole transcription regulatory elements, Txnip‐4 (860 bp) illustrated the full function of promoter with dynamic expression trend and inducible response to supplement of adenosine‐containing molecules.

As for cultured mammalian cells in vitro, especially CHO cells, Huong Le have explored and identified several genes, such as *Mmp12*, *Txnip*, and *Serpinf1*, with an upswing expression pattern in the stationary phase relative to the exponential phase, based on time‐series transcriptome data [12]. Compared with some strong promoter, such as CMV, SV40, or other viral promoters, this dynamic expression pattern could be used for expression of some cytotoxic proteins to avoid induction of ER stress responses and even apoptosis caused by incorrect protein folding and abnormal metabolism [29]. As shown in this work, the biomass of CHO‐pTxnip‐4‐rE2 cells could increase efficiently as same as parental CHO cells, even with the extra burden of expression of viral antigen under dynamic promoter. It could be speculated that the lower productivity of recombinant E2 protein driven by CMV promoter was ascribed to lower viable cell density because the expression of viral and cytotoxic antigen protein. With checking the two main substrates (glucose and glutamine) consumptions in the culture medium, continuous lactate accumulation during the whole culture process occurred in CHO‐pCMV‐rE2 A11 cells was probably contributed by glutaminolysis, which means a truncated cycle with oxidation of glutamine to the intermediate malic acid followed by conversion into pyruvate and finally into lactate [30].

Since recombinant CSFV E2 protein has been efficiently expressed under Txnip promoter with random integration of exogenous expression vector in CHO cells, specific transgene integration of rE2 coding sequences at the downstream of endogenous Txnip promoter has been performed in our laboratory by CRISPR‐Cas9 system, showing the same dynamic expression pattern in CHO‐K1 cells (data not shown in this work). And even in porcine kidney (PK15) cells and swine testicle (ST) cells that are used as host cell line for CSFV replication, the same specific integration strategy for rE2 expression under endogenous Txnip promoter of swine has been achieved successfully as well. With the infection of CSFV on recombinant PK15 or ST cells, the expression of rE2 protein driven by swine endogenous Txnip promoter could also been accelerated by introduction of inflammasome caused by virus infection and replication in host cells, since E2 and other non‐structure viral proteins had been tested with the trigger of NLRP3 inflammasome in infected host cells [31, 32].

Glucose uptake and utilization are highly regulated to maintain normal physiology and eukaryotic cells cultured in vitro employ diverse mechanisms to sense glucose and regulate the expression of different genes evolved in basic metabolism. Although it has been confirmed that the induction of Txnip expression by adenosine‐containing molecules is a glucose‐dependent manner, the detailed mechanisms and possible regulatory strategies for practical use remain elusive and need more investigation.

## CONFLICT OF INTEREST

The authors have declared no conflicts of interest.
